# Influence of Vigorous Physical Activity on Structure and Function of the Cardiovascular System in Young Athletes—The MuCAYA-Study

**DOI:** 10.3389/fcvm.2019.00148

**Published:** 2019-10-09

**Authors:** Lisa Baumgartner, Thorsten Schulz, Renate Oberhoffer, Heidi Weberruß

**Affiliations:** Institute of Preventive Pediatrics, TUM Department of Sport and Health Science, Technical University of Munich, Munich, Germany

**Keywords:** intima-media thickness, vascular stiffness, junior athletes, vascular adaptation, athlete's artery

## Abstract

**Objective:** Moderate physical activity (PA) is associated with a reduced risk to develop cardiovascular disease. However, junior athletes exercise between 10 and 20 h a week with intensities exceeding moderate levels by far. In this regard, the cardiovascular system has to increase its work five to six times compared to moderate intensities. This may result in potentially pathological adaptations of the cardiovascular system. The underlying process of vascular adaptations to exercise is yet not fully understood and hardly investigated in junior athletes. An increased blood pressure and pulse wave velocity, ventricular hypertrophy, arrhythmia, and even sudden cardiac death (SCD) has been reported in adult athletes. Studies, examining the cardiovascular system in children, its association to intensity and type of exercise, are rare. Therefore, we present the study protocol of a prospective cross-sectional study that investigates the influence of PA on the cardiovascular system in young athletes.

**Methods and Design:** Children and adolescents, 7–18 years, presenting for their annual pre-participation screening at the Institute of Preventive Pediatrics, Faculty of Sports and Health Sciences, Technical University of Munich (TUM), are examined in this prospective cross-sectional study. Vascular parameters measured by ultrasound are carotid intima-media thickness (cIMT), vascular stiffness (AC, Ep, β, PWV β), and the vascular diameter (D) to calculate the IMT:Diameter-Ratio (IDR). Cardiac function is evaluated by a 12-lead ECG, and echocardiographic parameters (end-diastolic left ventricular diameter, left ventricular diastolic posterior wall thickness, diastolic septal thickness, left ventricular mass and relative wall thickness, ejection fraction, and shortening fraction). A cardiopulmonary exercise test is performed on a bicycle ergometer, muscular strength is assessed with the handgrip test, and physical activity with the MoMo questionnaire.

**Discussion:** It is essential to follow young athletes over the course of their career in order to detect pathophysiological changes in the myocardium as soon as possible. If these changes are preceded or followed by changes in vascular structure and function is not known yet. Therefore, we present the study protocol of the Munich Cardiovascular adaptations in young athletes study (MuCAYA-Study) which investigates the association between vascular and cardiac adaptation to intensive exercise in junior athletes.

## Background

There is no doubt about positive effects of physical activity (PA) on the human body. Physically active persons reduce their risk of developing type 2 diabetes mellitus, hypertension, and cardiovascular disease (1). Prevalence of atherosclerosis is reduced as well as mortality of cardiovascular disease (2–7). Studies that describe positive effects of physical fitness focus on moderate exercise intensity in children and adults (7). According to WHO recommendations on physical activity (8) children should exercise 60 min at moderate intensity per day with short bouts of anaerobic intensities. Exercises to strengthen muscles and bones should be performed three times a week (8). However, young competitive athletes train between 10 and 20 h per week with intensities exceeding WHO recommendations by far (9). In this regard, the cardiovascular system has to increase its work five to six times, compared to moderate intensity levels (7), which potentially leads to adverse adaptations of the cardiovascular system.

The term “athlete's heart” describes a non-pathological electrophysiological, structural, and functional myocardial adaptation in response to continuous training stimuli in adults. Athletes present 10–15% increased left and right ventricles and a 10–20% increased left ventricular wall thickness. Functionally, an increased stroke volume and improved cardiac filling in diastole, improved capillary conductivity, and oxidative capacity of the skeletal muscle can be observed in athletes (7). Myocardiac cells adapt to regular PA according to the underlying stimulus (endurance or strength training). Endurance activities like long distance running or swimming reduce peripheral vascular resistance and systolic blood pressure (SBP), and increase cardiac stroke volume and output. As a result, myocardial oxygen consumption is reduced. Strength training increases myocardial oxygen consumption, heart rate, blood pressure, peripheral resistance, and stroke volume to a smaller extent (10).

Contrary to positive effects of PA, maladaptations of the cardiovascular system may occur, sudden cardiac death (SCD) is the most severe one (7, 11, 12). In 46 endurance athletes, mean age of 31 years, 18 cases of severe cardiac arrhythmia and 9 cases of SCD were described (13). The MARC study (Measuring Athlete's Risk of Cardiovascular Events) identified coronary sclerosis using cardio CT in almost 19% of athletes (>45 years) who had been asymptomatic, had a low cardiovascular risk profile according to the Systematic Coronary Risk Evaluation (SCORE) and an asymptomatic stress ECG (14).

Characteristic myocardial adaptations to intensive exercise training such as bradycardia, early repolarization, atrial dilatation, and ventricular hypertrophy are also seen in young athletes (15). In an American long-term survey, 1,866 cases of SCD or cardiac arrest were reported in the period between 1980 and 2006 (16). In 56% SCD was caused by cardiovascular disease with hypertrophic cardiomyopathy (HOCM) as predominant diagnosis (36%) (16). In competitive athletes (12–17 years), SCD incidence was highest with 1.17 cases per 100 000 athletes per year (17) with male athletes bearing double the risk than female athletes (18). Regarding these results, it is of high importance to follow young athletes over the course of their career to detect pathophysiological changes in the myocardium as soon as possible (19).

In addition to cardiac adaptations to PA, there is evidence that PA also has an influence on the vascular system. Green, Spence, Rowley, Thijssen and Naylor (20) postulate the concept of an “athlete's artery.” They hypothesize that persistently increased PA leads to an increase in diameter and reduction in wall thickness. Heffernan (21) suggests that this vascular remodeling is due to previous cardiac remodeling and triggered by high training intensities. They describe the following cycle: persistently increased cardiac output leads to an adverse effect on the endothelium, an increase in vascular stiffness and consequently end-organ damage of the heart. Hereby SCD risk is increased dramatically.

In young, normally active adults, Boreham et al. (22) found an inverse relationship between arterial vascular stiffness and cardiorespiratory fitness pointing toward reduced stiffness measures in athletes with higher maximum oxygen uptake (VO_2max_, *r* = −0.21, *p* < 0.001) and PA. Denham et al. (12) report lower vascular stiffness in moderately active men (*r* = −0.62, *p* < 0.001). In contrast, among competitive athletes, exercising at very high intensities, and control persons, negative adaptations to increased training intensities were found. Vlachopoulos et al. (23) report significantly higher blood pressure values and higher pulse wave velocity (PWV) in marathon runners (SBP 113 ± 15 mmHg vs. 102 ± 11 mmHg, diastolic blood pressure, DBP 79 ± 10 mmHg vs. 72 ± 9 mmHg, *p* < 0.001, and PWV 6.89 m/s vs. 6.33 m/s, *p* < 0.01). Abergel et al. (24) showed a 13% higher intima-media thickness (IMT) for professional road cyclists. Schmidt-Trucksäss et al. (25) observed an increased IMT of the femoral artery in endurance and strength-trained athletes. All results refer to comparisons with a control group.

In children, cIMT (carotid IMT) and arterial stiffness are inversely correlated with cardiorespiratory fitness and physical activity (26–29). In contrast to these positive adaptations, Kim et al. (30) observed higher vascular stiffness in young adult American football players. Feairheller et al. (31) report a higher carotid IMT and larger diameter of the brachial artery. In youth, there are no studies examining possible adaptation mechanisms caused by intensive exercise training. Furthermore, the influence of different training stimuli (type of sport, training frequency, and intensity) on the cardiovascular system has not been sufficiently investigated.

In summary, there is no consensus on the effect of intensive exercise on the vascular system. Studies show an influence of aerobic endurance training to vascular properties (20, 32, 33). However, the concept of the “athlete's artery” is not yet completely understood and only examined to a small extent.

Therefore, this prospective cross-sectional study investigates the influence of PA on the cardiovascular system in young athletes. For this purpose, the structure and function of the vascular system and myocardium are examined as well as the association to intensive exercise training. Confounding variables are types of PA (endurance, muscular endurance, strength), cardiopulmonary fitness, muscular strength, and training stimuli (frequency and intensity).

## Methods

### Design and Participants

Children and adolescents, 7–18 years, presenting for their annual pre-participation screening, are enrolled in this prospective cross-sectional study over the course of two years. Each year, approximately *n* = 250 children and adolescents are examined at the Institute of Preventive Pediatrics, Faculty of Sports and Health Sciences, Technical University of Munich (TUM).

The following inclusion criteria are applied:

- Age 7–18 years.
- Informed consent by children and/or legal guardians.
- No acute infection.
- No acute orthopedic injury.
- Medical clearance for cardiopulmonary exercise testing.

The study is in compliance with the Declaration of Helsinki and is approved by the TUM ethics committee (project number: 301/18 S). Informed consent is contained of all children and/or their legal guardians.

### Measurements

#### Parameters of Vascular Adaptation

cIMT as structural vessel parameter is measured after 10–15 min rest in supine position at the far wall of the common carotid artery with the head turned 45° opposite to the site being investigated. cIMT is examined 1 cm proximal to the bifurcation at the end of diastole at two angles on the right side (120°/150°) and two angles on the left side (210°/240°). Measurements are performed in B-mode ultrasound according to the guidelines of the Association for European Paediatric and Congenital Cardiology (AEPC) (34). Using the eTracking method (ET, M-mode), vascular stiffness, and common carotid artery diameter is measured in the same position, two times on the right and two times on the left side (150°/210°). Parameters of vascular stiffness are arterial compliance (AC), elastic modulus (Ep), stiffness index β (β), and local pulse wave velocity (PWV β), according to following formula (35):

(1) $$\begin{array}{c} \;AC=\;\pi \frac{\left(D_{max^{2}}-\;D_{min^{2}}\right)}{\text{4 }(BP_{max}-\;BP_{min})}\\ \;Ep=\;\frac{BP_{max}-\;BP_{min}}{\frac{D_{max}-\;D_{min}}{D_{min}}}\\ \;\beta =\;\frac{ln\frac{BP_{max}}{BP_{min}}}{\frac{D_{max}-\;D_{min}}{D_{min}}}\\ PWV\beta =\;\sqrt{\frac{\beta^{*}BP_{min}}{2\rho }} \end{array}$$

Vascular diameter (D) is measured at diastole, to calculate IMT:Diameter-Ratio (IDR) as parameter of functional adaptation (eccentric vs. concentric hypertrophy) of the vessel wall (36). cIMT and distensibility parameters of young competitive athletes will be compared to healthy controls (37). All measurements are performed with the ProSound Alpha 7 ultrasound system (Hitachi Medical Systems GmbH, Wiesbaden, Germany).

#### Parameters of Cardiac Adaptation

End-diastolic left ventricular diameter, left ventricular diastolic posterior wall thickness, and diastolic septal thickness are well-established parameters to describe myocardial morphology. They are examined via ultrasound (GE VIVID 7 Dimension ultrasound system and GE ECHOPAD software, GE Healthcare, Horten, Norway) in a resting position with slightly lateral inclination by two-dimensional M-mode. Structural parameters are compared to reference values of Pettersen et al. (38). Left ventricular mass is calculated according to Devereux and Reichek (39) and left ventricular relative wall thickness according to Lang et al. (40). Functional parameters are ejection fraction and shortening fraction.

#### Physical Activity

The MoMo activity questionnaire is a comprehensive questionnaire that records PA in different settings: school, everyday activity, PA organized in a club and leisure time PA outside a club (41). All activities are defined by frequency and/or duration and intensity. Minutes of exercise per week and metabolic equivalent (MET) minutes per week are calculated for each item, which allows calculation of an overall activity-index. One MET refers to the amount of oxygen (O_2_) the body consumes sitting at rest. It equals 3.5 ml O_2_ per kg body mass per minute (42). Additionally, overall PA is calculated according to Prochaska et al. (43) with the distinction on meeting the WHO-Guideline (8) or not.

#### Muscular Strength

The hand grip test is applied to assess childrens' strength and the influence of strength on cardiovascular adaptation. In a seated position with upright upper body, shoulders adducted, and elbow flexed in 90°, participants push the hand grip dynamometer (SAEHAN Hydraulic Hand Dynamometer SH5001, SAEHAN Corporation, Masan, South Korea) at maximum strength, three times with the right and left hand, respectively (44).

#### Cardiovascular Basic Diagnostics

All participants undergo a 12-channel ECG at rest (CARDIOVIT CS-200 Office, SCHILLER AG, Baar, Switzerland). Peripheral and central SBP and DBP, peripheral and central pulse pressure (PP), and aortic PWV are measured after a 5-min resting period using the Mobil-O-Graph (Mobil-O-Graph, IEM, Stolberg, Germany). The cuff (size according to upper arm circumference) is placed on the left arm.

#### Cardiopulmonary Fitness

VO_2max_ is determined by cardiopulmonary exercise testing (CPET, ramp protocol; Ergostik, Geratherm Respiratory GmbH, Bad Kissingen, Germany) on a bicycle ergometer (Lode Corival, Lode BV, Groningen, The Netherlands) plus 12 lead ECG. Relative maximum load (W/kg) and relative VO_2max_ (ml O_2_/min/kg) are calculated to determine participants' exercise capacity. Ventilation thresholds (VT1 and VT2) are defined by the V-slope method (45).

#### Personal and Family History

The personal history includes past and present illnesses and injuries, vaccination status, alcohol consumption, smoking cigarettes, drug use, psychological status, as well as girls' menarche, menstruation cycle and HPV vaccine. Family history includes past and current occurrence of cardiovascular disease, SCD, unclear death, metabolism disorders, diabetes mellitus, psychiatric disorders, malignancies, and presence of Marfan syndrome. In addition, exercise-related symptoms, such as dyspnoea, dizziness/syncope, palpitations or pain, are inquired.

#### Laboratory Chemical Analysis

Venous blood samples include the following parameters: hemoglobin, erythrocytes, hematocrit, MCV, MCH, MCHC, erythrocyte distribution, platelets, leukocytes, creatinine, GPT, GOT, uric acid, cholesterol, and CRP, to exclude infections, inflammatory diseases or anemia or increased blood lipid levels.

### Sample Size Calculation

A sample of *n* = 252 athletes is needed per year to detect a mean difference in cIMT of 0.05 mm, given a confidence interval of 95% and statistical power of 80%. The variation in this population is 0.04 mm. The difference in cIMT of 0.05 mm indicates the difference between median cIMT (0.46 mm) defined at the 50th percentile and an abnormal increased cIMT (0.51 mm) above the 95th percentile.

### Statistical Analyzes

Differences between structural and functional parameters of the cardiovascular system will be compared to age- and sex-specific reference values (37–39). Linear regression analyses are performed to investigate the association between VO_2max_ and training stimuli (frequency, intensity) to changes of the cardiovascular system and to analyze a correlation between arterial and myocardial adaptations, relative to the type of exercise (frequency, intensity). Dependent parameters are arterial structure and function as well as myocardial structure and function. Independent parameters are VO_2max_ and METs per week. Results are controlled for age, sex, BMI, BP, dietary behavior, blood cholesterol levels, and smoking.

## Discussion

This study aims to investigate the effect of PA on vascular and cardiac structure and function in young athletes. Furthermore, the influence of different types of PA (endurance, muscular endurance, strength), cardiopulmonary fitness, and training stimuli (frequency, intensity) on arterial structure and function are investigated, as well as the correlation of arterial structure and function to the structure and function of the heart.

### Cardiac Adaptation to PA

Cardiac remodeling is the process of changes of the myocardium. It is caused by hemodynamic pressure and volume loads (increased heart rate and ejection fraction, and increased blood pressure) and by biochemical mediators such as endothelin, cytokines, nitrogen, and oxidative stress by radical oxygen species (46). Hemodynamic influences stimulate cardiomyocytes to increase the expression of sarcomeres. Several studies have shown cardiac adaptations to PA in adult athletes (24, 47–50). Sharma et al. (7) consider too intensive exercise training as multifactorial risk factor in the course of an athlete's life that only becomes symptomatic in adulthood. The authors mentioned a higher risk to develop dilated cardiomyopathy in athletes with an enlarged left ventricle and lower ejection fraction. Makan et al. (51) compared 900 adolescent athletes (15.7 ± 1.2 years, endurance, kickback, and team sports) with 250 healthy controls. They showed an enlargement of the left ventricle in 18% of athletes (>54 mm) with normal systolic and diastolic function. Sharma et al. (19) studied the wall thickness of the left ventricle within the same collective (*n* = 720 athletes) and found an increased wall thickness in athletes compared to controls (9.5 ± 1.7 mm vs. 8.4 ± 1.4 mm, *p* < 0.01). In addition, 5% of participants had abnormal wall thickness values and showed an enlarged left ventricle (54.4 ± 2.1 mm). As highest values were observed in rowers, authors assumed a combination of intensive isometric and isotonic exercise that triggers hypertrophy of the left ventricle (19). In both studies, authors hypothesize an initially physiological adaptation of the myocardium that converts into a pathological adaptation.

The cardiovascular system is very sensitive to mechanical stimuli, such as an increased heart rate, increased ventricular filling, and increased arterial shear stress. This can also lead to adverse adaptations (52). In general, endurance training is considered to bear a positive effect on the myocardium. Strength training, on the contrary, has a potentially negative effect due to excessive pressure loads (53, 54). Heidbuchel et al. (13) do not differentiate between endurance and strength training, but consider intensive training bouts and/or recovery periods between training sessions that are too short, as one potential explanation for negative adaptations of the cardiovascular system. Adequate exercise training with an appropriate recovery time, on the other hand, may lead to a positive adaptation of the cardiovascular structure and function (55).

### Vascular Adaptation to PA

Green et al. (20) suspect increased blood pressure levels as trigger for changes in vascular structure and function. These changes are responsible for functional physiological as well as pathological processes. Bertovic et al. (32) observed increased vascular stiffness caused by blood pressure peaks during strength training. As stimulus, they consider a change in the proportion of smooth muscle cells in the intima layer and a relative increase in the ratio of collagen to elastin in the media layer. As intensive endurance exercise also causes peaks in blood pressure, this could be a possible trigger for pathological adaptations of the vascular structure, too.

Increased inflammatory markers after intensive exercise could also have an impact on blood vessels. Increased leukocytes and CRP were found in athletes after a marathon race and associated with an increase in vascular stiffness (20, 56, 57). Sharma et al. (7) refer to the relationship between increased arterial shear stress and increased oxidative stress, which may cause the development of atherosclerosis. The consequence of atherosclerotic changes is, inter alia, increased vascular resistance with subsequently higher myocardial oxygen consumption. The consequence of increased vascular stiffness is an increased ventricular afterload, which leads to left ventricular hypertrophy and decreased cardiac perfusion during diastole (58). Whether the increased vascular stiffness causes cardiac changes or if increased vascular stiffness is the result of previous myocardiac adaptations has not yet been investigated.

Positive adaptations due to exercise can be induced by increased shear stress and the associated release of vasoactive substances such as NO (12, 20, 26, 59). Furthermore, moderate endurance exercise stimulates endothelial progenitor cells that positively influence endothelial function (59–61). Moderate aerobic endurance exercise has a positive effect on blood pressure levels and is recommended as non-pharmacological therapy to treat arterial hypertension (62). Vasodilating agents that are released during exercise are only slowly reduced at the end of moderate exercise. They result in low blood pressure levels (63, 64). The mechanism of this effect, called post-exercise hypotension (PEH), can last up to 10 h, with positive effects (increased elasticity or decreased rigidity) on the vascular system (65, 66).

In general, functional and structural changes are less pronounced in adolescent athletes due to their younger training history than in adults (19, 51). At this age, pathophysiological changes could still be counteracted and therefore prevented by adequate training control.

### Interaction of Cardiac and Vascular Adaptation to PA

In line with the definition of the athlete's heart, it is questionable in which way the vascular system adapts to training loads and whether there is a physiological relationship between cardiac and vascular adaptation (20, 67). Bell et al. (68) report an association between femoral artery stiffness and Global Longitudinal Strain (GLS) in adults without cardiovascular disease in the Framingham Heart Study—the higher the vessel stiffness, the higher and thus the worse was GLS. Based on the literature, this effect is intensified by intensive exercise. The association between vascular and cardiac adaptations to PA in youth has not been fully studied. In addition, it is unclear whether the known adaptation phenomena of the myocardium precedes an adaptation of the vascular system or whether the vascular system subsequently adapts to changes of the myocardium.

Several studies demonstrate a U- or J-shaped curve of PA and health. Initially, physical activity is positively associated with better health status up to the vertex, which reflects the turning point with higher risk for cardiovascular events underneath and above a healthy threshold of PA in adults (69–71). The question therefore arises, whether this negative effect of exceptionally high volume of PA manifests itself in youth. In addition, it may therefore be possible that prolonged and intensive PA impairs vascular health in young athletes. In this context, pathophysiological reactions as result of intensive PA should be investigated in relation to the type, intensity, and duration of PA.

## Limitations

This study is a first step towards an analysis of cardiac and vascular structure and function in junior athletes. It will provide cross-sectional data as basic research for future longitudinal studies. The age-range covered is from 7 to 18 years and includes participants within different levels of their physiological, neurological, and mental development. It covers the age-range before, during, and after puberty which has an impact on hormonal status and thus on physical components. To account for this differences, participants will be compared to age- and sex-specific reference values and within their age-groups.

We collect data on participants' training history to account for the time span they've been involved in competitive sports, to differentiate between shorter and longer exposure to physical training. What we cannot account for with this data is how participants respond to physical stimuli, as there will be athletes being predominantly disposed to muscular and cardiovascular adaptation than others. Genetic factors are not involved in the study at this point but could be one major aspect of future work as well as more laboratory parameters. Family history is assessed in the medical interview and will be reviewed for a history of cardiac disease or other health issues. This part could also be extended toward a family training history.

## Conclusion

The positive influence of physical activity on the cardiovascular system is affected by negative adaptations of intensive training stimuli and years of intensive endurance exercise in adults. This is reflected by changes in myocardial morphology and myocardial function as well as vascular structure and vascular function. In young athletes, increased wall thickness and increased lumen of the left ventricle were observed. Studies investigating the influence of intensive physical activity on the structure and function of the vascular system in young athletes do not yet exist. In children and adolescents studies show an association between moderate physical activity and positively altered structural and functional vascular parameters - however, these studies refer to athletes with normal levels of activity (according to the WHO recommendation). The role of type of sport, exercise frequency, and exercise intensity in the adaptation process is still unclear in adults and young athletes. Additionally, the long-term effects of this adaptation have not been sufficiently investigated (20, 72). This raises the question whether there is too much exercise from a cardiovascular point of view and whether a relationship between vascular function and vascular structure as well as myocardial function and myocardial morphology can be observed in young athletes.

## Ethics Statement

The studies involving human participants were reviewed and approved by Ethics committee of the Technical University of Munich (project number: 301/18 S). Written informed consent to participate in this study was provided by the participants and/or legal guardian.

## Author Contributions

LB and HW were involved in study conception and drafting the manuscript. TS and RO were involved in study conception and critically reviewed the manuscript. All authors have read and approved the final version of this manuscript.

### Conflict of Interest

The authors declare that the research was conducted in the absence of any commercial or financial relationships that could be construed as a potential conflict of interest.

## Abbreviations

AC: arterial compliance
AEPC: Association for European Paediatric and Congenital Cardiology
cIMT: carotid intima-media thickness
CPET: cardiopulmonary exercise testing
D: vascular diameter
DBP: diastolic blood pressure
Ep: elastic modulus
GLS: global longitudinal strain
HOCM: hypertrophic cardiomyopathy
IDR: IMT:Diameter-Ratio
IMT: intima media thickness
MARC study: Measuring Athlete's Risk of Cardiovascular Events
MuCAYA-Study: Munich Cardiovascular Adaptation in Young Athletes Study
PA: Physical activity
PEH: post exercise hypotension
PWV: pulse wave velocity
PWV β: pulse wave velocity β
SBP: systolic blood pressure
SCD: sudden cardiac death
SCORE: Systematic Coronary Risk Evaluation
TUM: Technical University of Munich
VO_2max_: maximum oxygen uptake
β: stiffness index β.
